# Redox active plant phenolic, acetosyringone, for electrogenetic signaling

**DOI:** 10.1038/s41598-024-60191-7

**Published:** 2024-04-26

**Authors:** Fauziah Rahma Zakaria, Chen-Yu Chen, Jinyang Li, Sally Wang, Gregory F. Payne, William E. Bentley

**Affiliations:** 1 Fischell Department of Bioengineering, University of Maryland, College Park, MD USA; 2 Institute for Bioscience and Biotechnology Research, Rockville, MD USA; 3 Robert E. Fischell Institute for Biomedical Devices, University of Maryland, College Park, MD USA; 4 Division of Biology and Biological Engineering, California Institute of Technology, Pasadena, CA USA

**Keywords:** Chemical engineering, Biotechnology, Expression systems, Metabolic engineering

## Abstract

Redox is a unique, programmable modality capable of bridging communication between biology and electronics. Previous studies have shown that the *E. coli* redox-responsive OxyRS regulon can be re-wired to accept electrochemically generated hydrogen peroxide (H_2_O_2_) as an inducer of gene expression. Here we report that the redox-active phenolic plant signaling molecule acetosyringone (AS) can also induce gene expression from the OxyRS regulon. AS must be oxidized, however, as the reduced state present under normal conditions cannot induce gene expression. Thus, AS serves as a “pro-signaling molecule” that can be activated by its oxidation—in our case by application of oxidizing potential to an electrode. We show that the OxyRS regulon is not induced electrochemically if the imposed electrode potential is in the mid-physiological range. Electronically sliding the applied potential to either oxidative or reductive extremes induces this regulon but through different mechanisms: reduction of O_2_ to form H_2_O_2_ or oxidation of AS. Fundamentally, this work reinforces the emerging concept that redox signaling depends more on molecular activities than molecular structure. From an applications perspective, the creation of an electronically programmed “pro-signal” dramatically expands the toolbox for electronic control of biological responses in microbes, including in complex environments, cell-based materials, and biomanufacturing.

## Introduction

Integrating electronic networks with biological systems generates powerful opportunities to reveal insights about complex natural systems^1^ and provides novel methods to confer precise control over such systems^2^. Connecting molecular communication networks to electronics is of emerging interest as it takes advantage of the well-established, simple, modular, and programmable nature of electronic devices. Facilitating electronic communication with biology allows for finely programmed behavior of responsive cells. Owing to the ubiquity of electronic devices, we believe that electrogenetic technologies, where electronics are used to control gene expression, can potentially revolutionize synthetic biology^2,3^ and bioelectronic technologies^4,5^. By developing the field of electrogenetics, designing gene circuits with increasingly complex behaviors will become more facile, expanding the capabilities of controlling living cells^6^.

A key challenge in connecting biology and electronics lies in their disparate communication modalities: molecular signals carry information based on structure on the cellular level, whereas the flow of electrons governs electronic communication. However, electronic and cellular circuits operate with similar principles and thus could have compatible signal processing^7,8^. Because redox activity is one of biology’s most prevalent signaling modalities^8^, redox activity provides a biologically natural way to interconvert between electronic and molecular, cellular systems. Microbes may be well-suited to convert redox signals into cellular pathway signals, as they contain a multitude of sensors that transduce redox signals into structural changes^9^. Electrogenetics may repurpose these redox sensors to control gene expression. For instance, redox-active diffusible molecules, or mediators, can transport electrons between an electrode and cells harboring redox-responsive promoters^10^.

Previous demonstrations using redox for electronic control of gene expression rewired redox-responsive regulons such as *soxR*, that is responsive to redox-cycling drugs^11^; *oxyR*, that is responsive to hydrogen peroxide^12^; and KEAP1 and NRF2, responsive to reactive oxygen species^13^. By applying an oxidative potential to mediators ferricyanide and pyocyanin, Tschirhart and others^14^ toggled bacterial gene expression that is regulated by transcriptional regulator SoxR. With this system, electronic inputs controlled phenotypes such as swimming^14^ and microbial signaling^13^, as well as CRISPR-based signal amplification and noise reduction^15^. For a second, distinct mechanism for electrogenetic control, electrode-generated hydrogen peroxide from the reduction of dissolved oxygen^16^ was used to electrogenetically modulate the *oxyR* regulon, enabling control of consortia composition^17,18^ and improvement of small molecule production in a co-culture^19^. Electrogenetic signaling is a newly emerging field, and the repertoire of electrochemical inducers and their respective genetic parts is limited. Development of new parts for the electrogenetic toolkit, such as redox-linked elements, will be crucial for designing more complex electronics-controlled genetic circuits, increasing the range of specificity, building bio-electronic devices, and connecting to environments rich in redox signaling.

That is, redox signaling is abundant in biology, in contexts including the gut microbiome^20^, soil rhizosphere^21^, and disease and inflammation^22^. Despite its ubiquitous nature, the mechanisms and networks of redox signaling remain poorly understood^23–25^, and the chemical tools for studying redox species often suffer from limitations^26,27^. Electrochemical tools offer a different approach for studying redox, and indeed have already shown promise by revealing new insights, through methods such as mediated electrochemical probing^28,29^.

The *oxyRS* regulon is a global stress response regulon that enables bacterial cells to adapt to and survive oxidative stresses, typically peroxides. Electronically controlling expression of the *oxyRS* regulon was recently demonstrated^30^ and involves the OxyR protein, which in its native state is reduced and inactive. When oxidized by hydrogen peroxide, OxyR undergoes a conformational shift owing to restructuring of disulfide switches^31^, binds with RNA polymerase, and positively regulates transcription of its dependent promoters, including the promoters for *oxyS* and *oxyR*^32^. We hypothesized that redox mediators in their oxidized state might also directly oxidize OxyR, which has a redox potential (E^0^) of − 185 millivolts^33^, or might indirectly lead to oxidized OxyR through the generation of peroxides that, in turn, restructure OxyR disulfide switches.

Here, we investigated redox mediator 1-(4-Hydroxy-3,5-dimethoxyphenyl)ethan-1-one (acetosyringone), a plant-derived phenolic signaling compound^34^ (E^0^ =  + 0.5 V^35^ vs. Ag/AgCl), for its ability to induce OxyR-regulated gene expression. Like many phenolic compounds, acetosyringone (AS) is produced by plant tissues in response to stress and pathogen infection^36^. The observation that AS contributes to a redox potential burst during the plant’s oxidative response to infection^37^ suggests that it acts as a redox-modulating agent affecting pathogen responses. Interestingly, oxidized AS was also demonstrated to mediate disulfide bond formation between cysteine molecules^38^ as well as the thiol groups of thiolated poly(ethylene glycol)^35,38^, providing a putative mechanism by which AS could interact with the thiol groups of the OxyR subunits and activate OxyR transcriptional responses if it were transported into the cells in an oxidative state or otherwise oxidized intracellularly. Such a phenomenon would indicate AS acting as a redox signal, in contrast to the well-studied AS signaling by molecular structure, as in the *vir* regulon. Interestingly, the two-component signal transduction process of *Agrobacterium tumefacians*^39^ consists of a membrane bound receptor (VirA), and its cognate transcriptional regulator (VirG); these have been transformed into *E. coli* enabling AS-activated gene expression^40^, but the oxidation state of the AS signal was not reported. Since AS in the native form is reduced we expect reduced AS is the activating signal. *E. coli* homologues to the VirA receptor and VirG transcriptional regulator have not been reported. Moreover, the *Agrobacterium* system does not involve OxyR-based disulfide switches.

In this work, we demonstrate electronic control of gene expression of the *oxyS* promoter by addition of oxidized acetosyringone, in addition to induction via production of hydrogen peroxide. That is, we show electrogenetic control of *oxyRS* by bolus addition of oxidized acetosyringone to reporter cells and by applying oxidative potential to a mixture of reporter cells and AS. We further demonstrate that distinct electrochemical mechanisms (oxidation of AS at oxidizing potentials, and the reduction reaction for H_2_O_2_ production at reducing potentials) can actuate a single promoter. That is, we show duality in the approach. By applying charge at + 0.5 V, one can oxidize AS and subsequently stimulate *oxyRS* gene expression. Transitioning towards negative (reduced) voltages has no effect, then transitioning further to a more reducing voltage (~ − 0.5 V) again stimulates expression via the identical promoter. This duality will enable more sophisticated means for electronically programming genetic circuits, potentially including real time measurement and control. Finally, we also show how dynamic application of these transient induction mechanisms can be overlaid to build complex cellular responses. In sum, our findings reveal that oxidized AS is a novel inducer of OxyR, opening up a richer set of possibilities for the prototypical stress-response mechanism and electrogenetics.

## Results

### Characterization of acetosyringone oxidation

**Figure 1:**
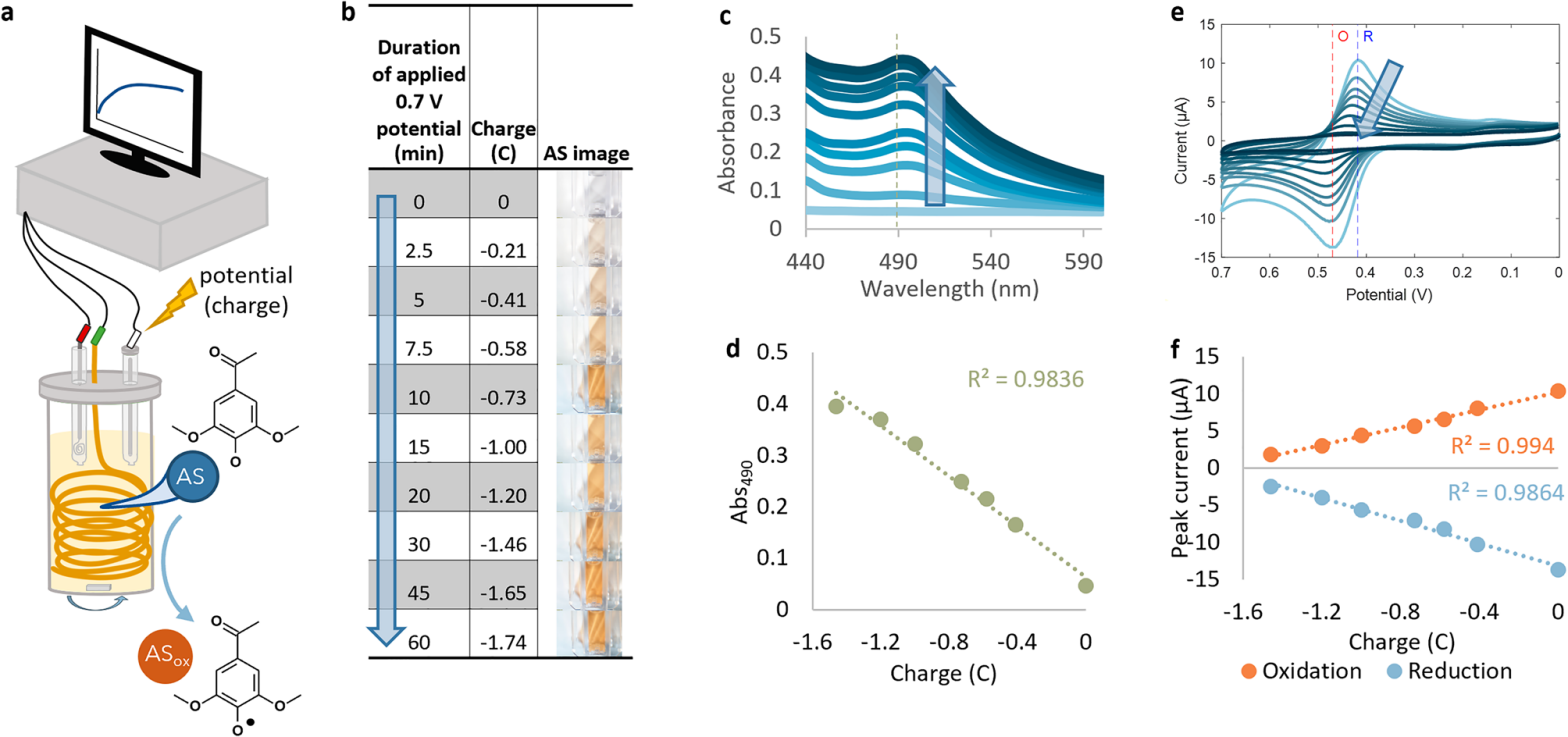
Acetosyringone (AS) electrochemical oxidation is robustly characterized. (**a**) Electrochemical setup for application of charge to AS solution. Gold wire working electrode, Ag/AgCl reference electrode, and platinum counter electrode (separated by a salt bridge) were submerged in AS solution and connected to a potentiostat. Accumulated charge, optical images of AS solution in a cuvette (**b**), absorbance spectra (**c**), and cyclic voltammogram (CV, **e**) of 2 mM AS which was charged (oxidized) with + 0.7 V for 0–60 min. Images are taken immediately post applied charge using Olympus MVX10 Macro Zoom Fluorescence Microscope and cellSens Standard 2.1. Reduction peak (‘R’) and oxidation peak (‘O’) are marked on CV. Arrows indicate application of charge to AS for a longer duration of time. (**d**) Correlation of applied charge to absorbance at 490 nm and (**f**) to currents at oxidation and reduction peaks. Schematic (**a**) was generated using Microsoft PowerPoint Version 2401 and PubChem Sketcher V2.4. (**e**) was generated in Matlab R2020a. All other plots were generated in Microsoft Excel Version 2401.

We oxidized a 2 mM solution of acetosyringone (AS) in phosphate buffer (PB) by applying a positive potential to the solution in a half-cell setup^41^ (Fig. 1a). Applying a potential of + 0.7 V—higher than the E^0^ of AS—for 0 to 60 min was equivalent to application of 0 to -1.74 coulombs of charge. This gradually turned the AS solution from colorless to brownish-orange, indicating its oxidation^42,43^ (Fig. 1b) and increasing levels of AS oxidation were characterized spectrophotometrically with an increasing absorbance peak at 490 nm (Fig. 1c). Interestingly, the color change was linear with applied charge (Fig. 1d). AS oxidation was also characterized electrochemically using cyclic voltammetry. When scanning from 0 to 0.7 V, AS had peak currents at 0.47 V (oxidation peak or E_pa_, peak anodic potential) and 0.417 V (reduction peak or E_pc_, peak cathodic potential), and both peak currents were attenuated with increasing application of oxidative charge (Fig. 1e). Again, current attenuation was linear with applied charge (Fig. 1f). Oxidation of AS when suspended in phosphate buffer (PB) at pH 7.4 can thus be characterized by absorbance at 490 nm, current at the oxidative peak, and current at the reductive peak, as each of these three metrics correlated to the applied potential.

### Oxidized acetosyringone imparts oxidative stress on *E. coli*

**Figure 2:**
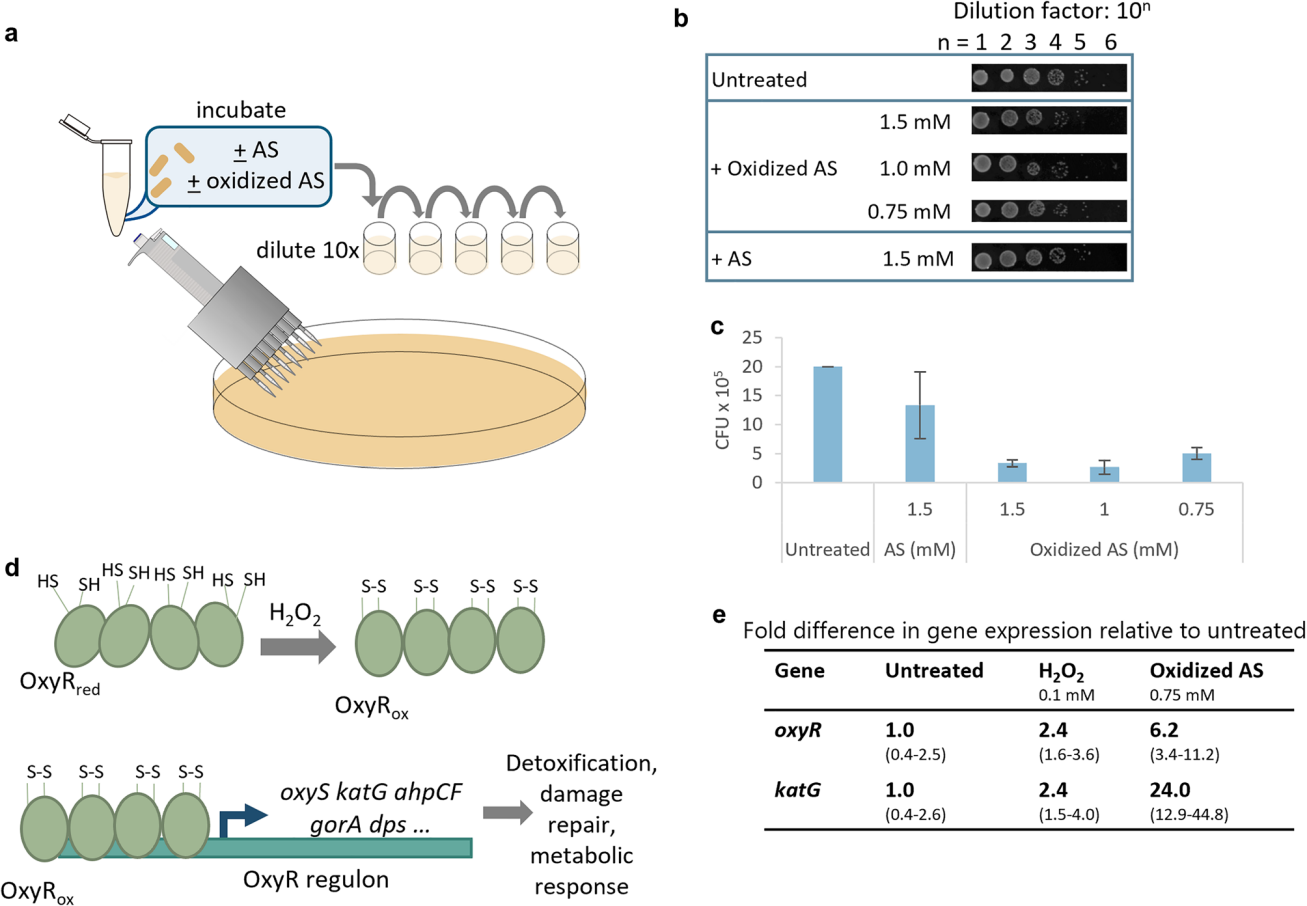
Oxidized acetosyringone elicits cellular oxidative stress response. (**a**) Scheme of colony count assay. (**b**) Colonies and (**c**) colony forming units (CFU) observed after cells were mixed with oxidized and reduced AS. Cells in LB were mixed with AS or phosphate buffer (PB, as a negative control), incubated for 1.5 h with shaking at 37 °C, and plated in 10× dilutions. Images were taken using an Amersham Imager 680. (**d**) The OxyR regulon in *E. coli*. The OxyR regulator is inactive in its reduced state. Upon oxidation by H_2_O_2_, oxidized OxyR binds to DNA at the promoter region of cognate genes and activates transcription. Adapted from Pomposiello and Demple^31^. (**e**) qPCR data showing effect of oxidized AS on cellular expression of stress-related genes *oxyR* and OxyR-regulated *katG*, with standard deviation of fold difference shown in parenthesis. Cells were grown to OD_600_ = 0.4, treated with the respective inducer, and pelleted at an OD_600_ of 0.8–0.9 for RNA extraction. Schematics (**a**) and (**d**) were generated in Microsoft PowerPoint Version 2401. (**c**) was generated in Microsoft Excel Version 2401.

Perhaps oxidized AS inhibits pathogen invasion of plants by increasing toxicity towards microbes^44^. While *E. coli* is not a typical plant pathogen, we performed a colony count assay after treating *E. coli* with PB, AS, and oxidized AS (Fig. 2a), and we found oxidized AS inhibited *E. coli* growth more strongly than AS in its natural (reduced) state (Fig. 2b,c), suggesting a negative impact on the cell’s metabolic state. We hypothesized that the OxyR hydrogen peroxide-responsive stress regulon might be involved in the cellular response to oxidized AS. The OxyR protein is intracellularly oxidized by hydrogen peroxide and activates transcription of genes encoding catalase (*katG*), glutaredoxin (*grxA*), small noncoding transcriptional regulator OxyS RNA (*oxyS*), and other responding molecules to the H_2_O_2_ stress (Fig. 2d). Indeed, qPCR revealed that oxidized AS upregulated expression of two OxyR-regulated genes, *oxyR* and *katG* (Fig. 2e). Upregulation of genes in the *oxyR* regulon by oxidized AS suggests the potential to use oxidized AS for synthetic gene expression through the OxyR-regulated *oxyS* promoter.

### Setting the redox state of acetosyringone enables control of gene expression

**Figure 3:**
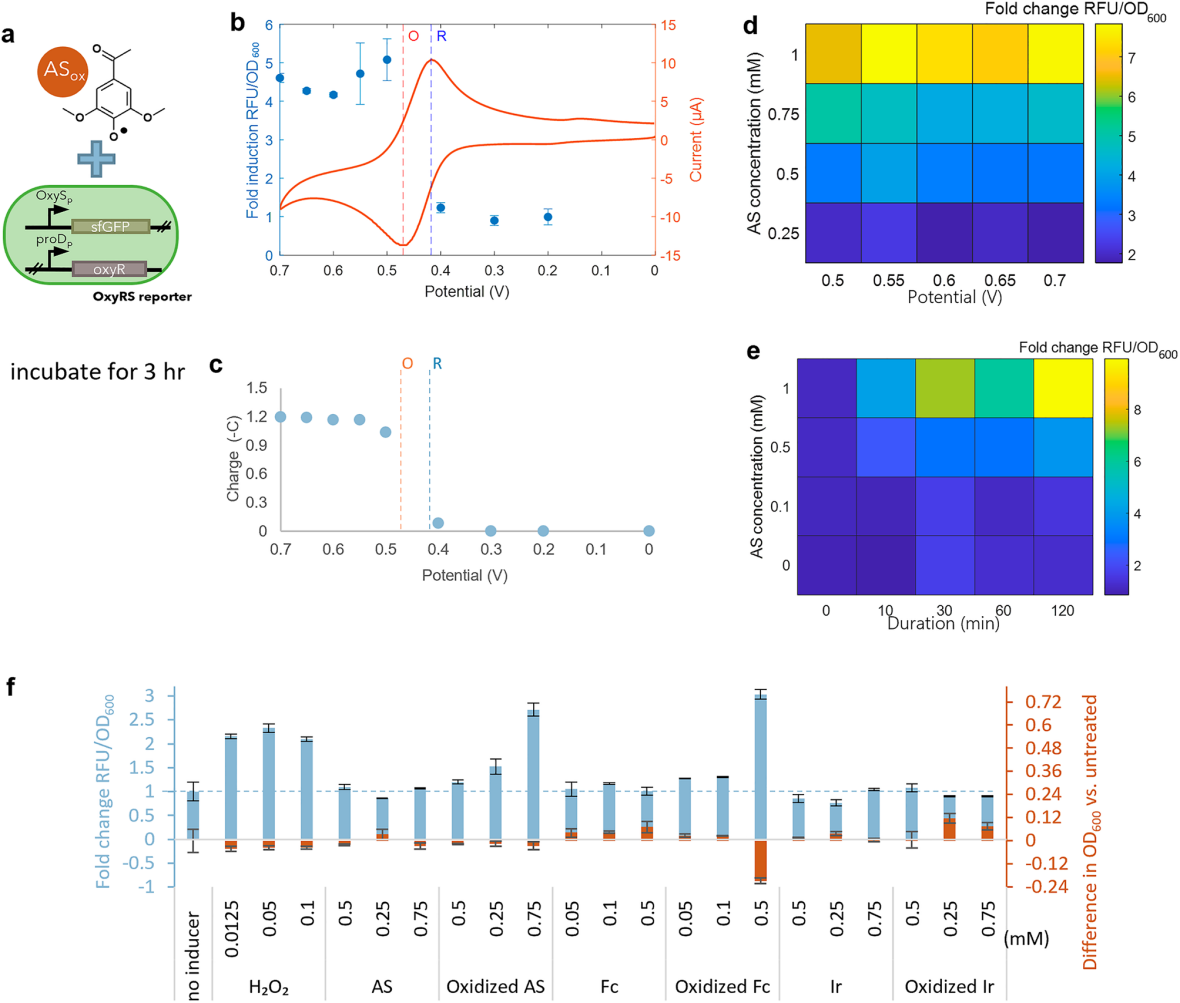
Oxidized acetosyringone (AS) induces the OxyRS promoter. (**a**) Scheme of experiment and gene circuit of OxyR reporter cells. (**b**) The indicated potentials were applied to AS for twenty minutes. OxyR reporter cells were mixed with 0.5 mM AS (to which the potential was applied) and incubated, and fluorescence was measured. Cyclic voltammogram shows oxidative (O) and reductive (R) peaks of AS. (**c**) Accumulated charge after applying indicated potentials to AS for 20 min. (**d**) AS was charged at varying potentials for 20 min and added to reporter cells at varying concentrations. Reporter cell fluorescence was measured. (**e**) Cell fluorescence resulting from induction by AS (oxidized for varying durations of time at + 0.7 V) at varying concentrations. (**f**) Fluorescence and final OD_600_ of cells treated with H_2_O_2_, AS, ferrocene (Fc), and iridium (Ir) in reduced and oxidized states. (**b**–**f**) show data after three hours of incubation; fluorescence is normalized to OD_600_ and reported as fold change relative to untreated cells. Schematic (**a**) was generated using Microsoft PowerPoint Version 2401 and PubChem Sketcher V2.4. (**b**), (**d**), and (**e**) were generated in Matlab R2020a. (**c**) and (**f**) were generated in Microsoft Excel Version 2401.

To investigate whether oxidized AS could induce expression via hydrogen peroxide-inducible OxyR, we used previously engineered *E. coli* OxyRS-sfGFP reporters which express sfGFP downstream of the *oxyS* promoter and OxyR downstream of the constitutive *proD* promoter^30^ (Fig. 3a). Applying to AS potentials below its E_pc_ had little effect on OxyRS-sfGFP reporters, while oxidizing AS by applying oxidizing potentials higher than its E_pa_ (0.47 V) induced sfGFP expression resulting in measurable green fluorescence (Fig. 3b). The charge accumulated while applying potential to AS served as a gate for induction of the *oxyS* promoter. Applying potentials below the E_pc_ of AS caused minimal accumulation of charge (< − 0.086 C), and the resulting AS did not induce gene expression. Applying oxidizing potentials caused charge accumulation over − 1.04 C, yielding oxidized acetosyringone which could induce the *oxyS* promoter (Fig. 3c). Expression levels were then shown to be modulated by the concentration of oxidized AS and the magnitude of potential applied to AS (Fig. 3d), with appreciable fluorescence induced only at higher concentrations and oxidizing potentials (> ~ 0.5 mM, >  ~ 0.5 V). We also found that expression level could be modulated by the duration of the applied oxidative potential (Fig. 3e), indicating that both the potential and charge duration applied to AS contribute toward accumulated charge and consequently the extent of AS oxidation. To compare the specificity of oxidized AS as a mediator inducing *oxyR* regulon expression, mediators ferrocene and iridium (E_0_ of + 0.25 and + 0.67^35^ vs Ag/AgCl, respectively) were also tested. Ferrocene and iridium were oxidized by application of + 0.3 V or + 0.9 V, respectively^35^, then added to OxyRS-sfGFP reporter cells in both the reduced and oxidized states. We also measured the final OD_600_ of the cultures to establish effects on cell growth. The two mediators at the concentration ranges tested either did not induce appreciable induction of the reporter cells and had little effect on growth or strongly induced expression but greatly inhibited growth (Fig. 3f), suggesting that oxidized AS selectively induces the *oxyS* promoter in a concentration dependent manner and at the same time has minimal effect on growth at concentrations leading to induced expression (i.e. < 750 μM).

### Distinct *oxyS* promoter induction dynamics by H_2_O_2_ and oxidized acetosyringone

**Figure 4:**
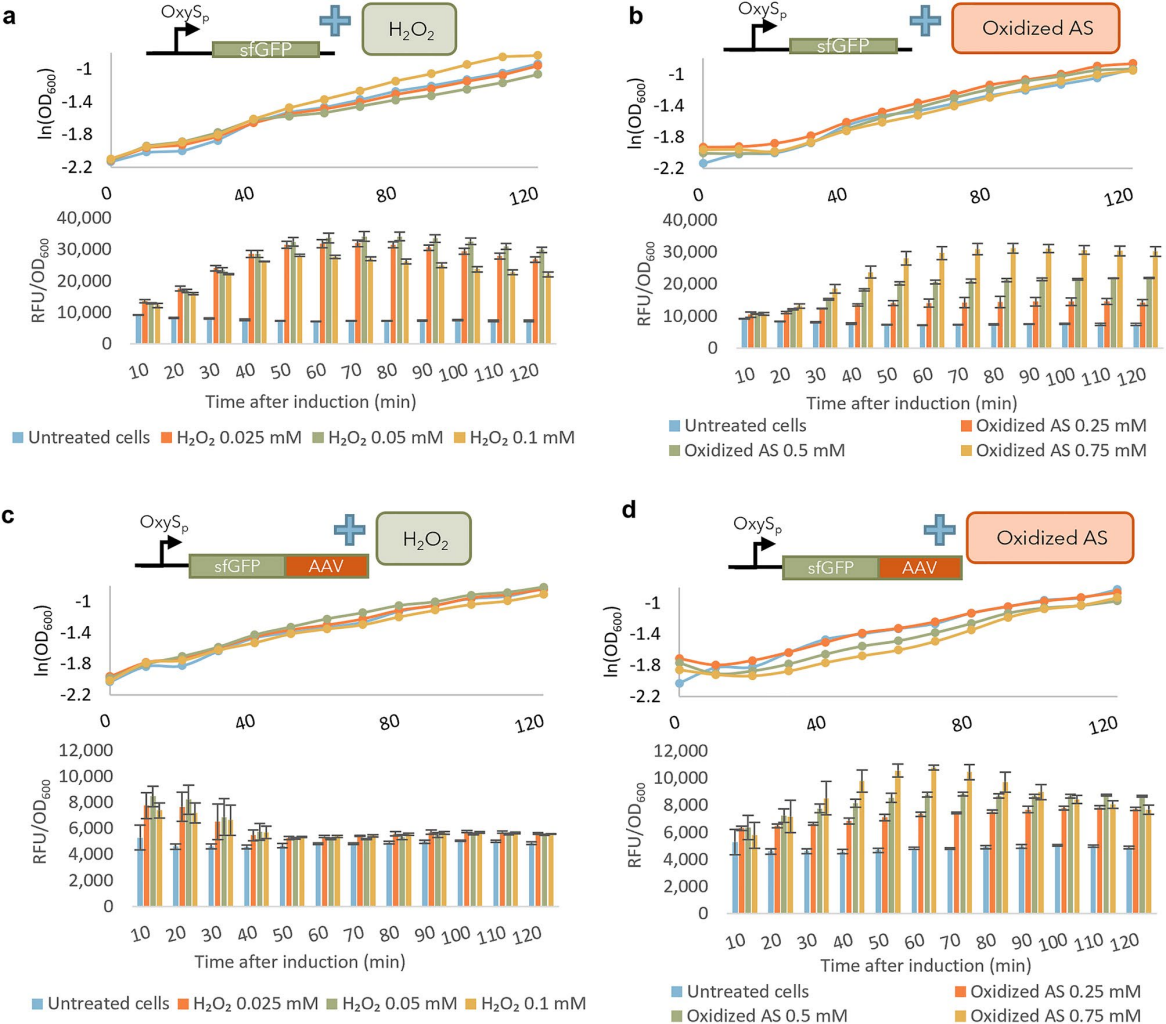
Oxidized AS elicits a distinct response from the OxyRS promoter. OD_600_ and fluorescence of cell reporters induced with H_2_O_2_ or with AS oxidized for 20 min at + 0.7 V. Cell reporters expressed sfGFP under control of the OxyRS promoter with (**a**,**b**) or without (**c**,**d**) a degradation tag (AAV). (**a**–**d**) show fluorescence normalized to OD_600_ and were generated in Microsoft Excel Version 2401. Schematics were generated in Microsoft Powerpoint Version 2401.

These results show that oxidized AS induces *oxyRS*, and while it is well-known that H_2_O_2_ also induces *oxyRS*, oxidized AS and H_2_O_2_ are electrochemically generated at dramatically different potentials. In Fig. 4, we found that both oxidized AS and hydrogen peroxide can induce expression through the OxyR-regulated *oxyS* promoter, but the dynamic responses were quite distinct. Fluorescence from the OxyRS-sfGFP reporter induced by H_2_O_2_ started to rise immediately after addition of H_2_O_2_ and reached a peak 60–80 min afterwards (Fig. 4a). In contrast, sfGFP fluorescence induced by oxidized AS rose more slowly and leveled off after 70–80 min without a subsequent decrease (Fig. 4b). The pretreated cells in both cases were identically cultured hence similarly metabolically active. Fluorescence-activated cell sorting confirmed the distinct dynamics and extent of induction elicited by H_2_O_2_ and oxidized AS (Supplementary Fig. S1). The different responses to H_2_O_2_ and oxidized AS were further highlighted by using OxyRS-sfGFP-AAV reporter cells in which sfGFP was fused with an ssRA^45^ degradation tag on its C-terminus. In this way, the sfGFP measurement is more reflective of the rate of generation as opposed to the absolute level. H_2_O_2_-induced fluorescence in the OxyRS-sfGFP-AAV reporters rose rapidly immediately after H_2_O_2_ addition, peaked after 20 min, and subsequently fell to the earlier uninduced reporter cell levels (Fig. 4c). These data indicated that the response to hydrogen peroxide addition was strong and finite in time, likely owing to the degradation of inducer, H_2_O_2_, at these cell densities^46^. Oxidized AS, on the other hand, induced a slower rise and a more sustained fluorescence (Fig. 4d), indicating continued oxidative stress (and expression) throughout.

We found minimal differences in growth rate between the different induction methods at the selected H_2_O_2_ and oxidized AS concentrations. Further, we performed a simple dynamic analysis of this system, calculating a zeroth order maximum rate expression and a first-order GFP decay rate spanning the appropriate times in Fig. 4a–d (Supplementary Fig. S2 and Supplementary Table S1). We found the maximum rate of synthesis was nearly identical for all hydrogen peroxide addition cases without a degradation tag (Fig. 4a) and the oxidized AS case at the 0.75 mM level (Fig. 4b). Interestingly, we also found nearly linear dependence of this maximum synthesis rate with AS concentration. We next found that the first-order degradation rate for the H_2_O_2_-induced sfGFP-AAV case (with the degradation tag) was roughly threefold larger than the largest degradation rate for the AS-induced sfGFP-AAV case (Supplementary Fig. S2 and Supplementary Table S1). This was somewhat surprising and suggested that the response to hydrogen peroxide elicited more proteolytic activity than the AS addition, although this was not tested. These results do show, however, that the responses of the cells to the different methods of induction were different.

### Electronic control of gene expression

**Figure 5:**
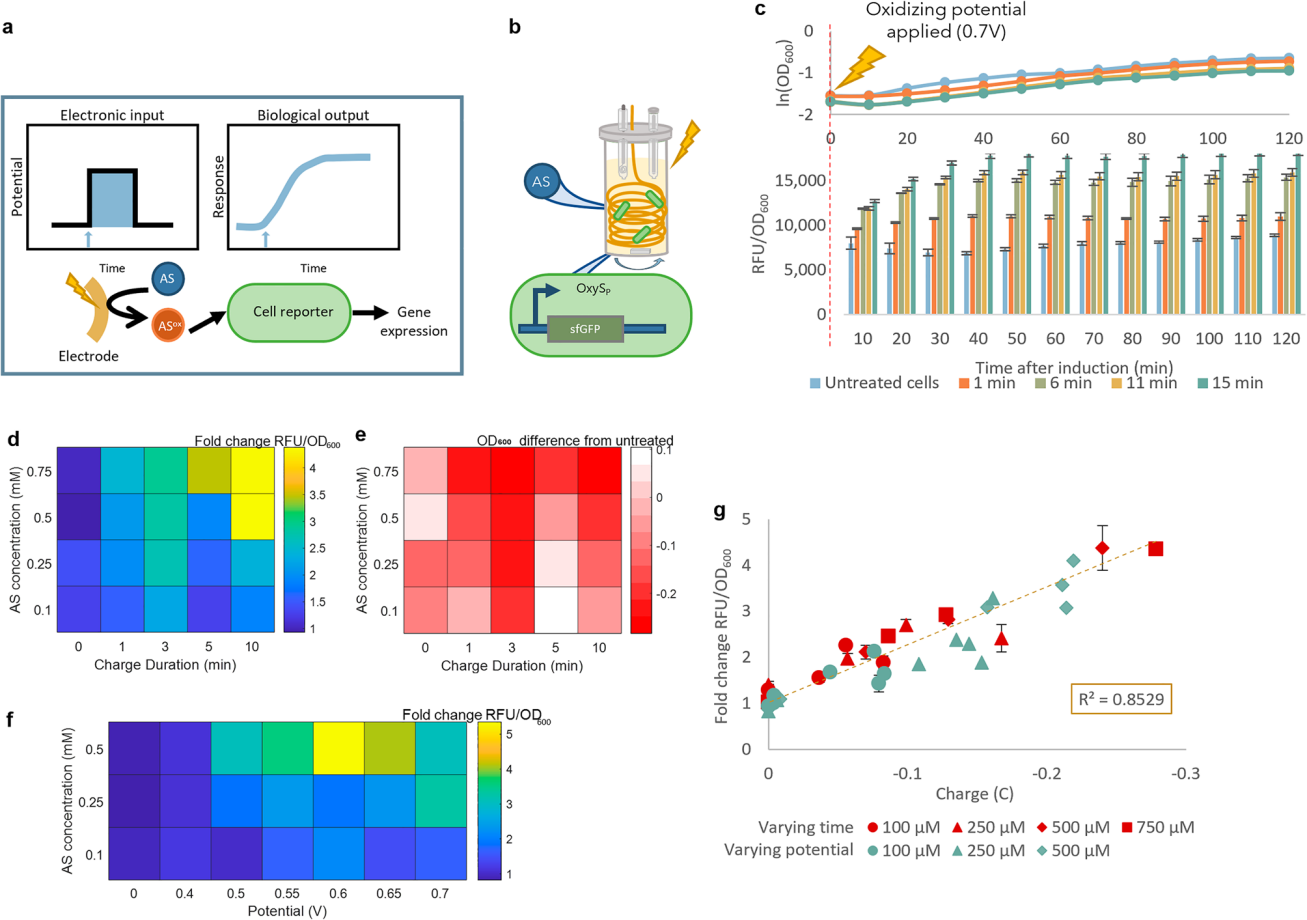
Acetosyringone (AS) can be applied for electronic control of cell fluorescence. (**a**) Direct electrogenetic control scheme. A potential applied through the gold electrode causes oxidation of AS in solution, which induces a cellular response in the cell reporters via the *oxyS* promoter. (**b**) Scheme of the electrochemical setup. A gold wire working electrode, Ag/AgCl reference electrode, and platinum counter (separated by a salt bridge) are submerged in a 2.4 mL mixture of liquid cell culture and AS. (**c**) OD_600_ and fluorescence of cell reporters induced by direct electrogenetic control. 100 μM AS was added to reporter cells, and an oxidative potential of + 0.7 V was applied for the indicated duration. Fluorescence is reported as the moving average of fluorescence over 30 min with 3 technical replicates. (**d**) Fluorescence of reporter cells after addition of AS at varying concentrations and application of + 0.7 V for varying durations of time. Fluorescence was normalized to that of untreated cells, and (**e**) the difference in OD_600_ between treated and untreated cells three hours after induction is shown. (**f**) Fluorescence of reporter cells, normalized to untreated, after addition of AS at varying concentrations and application of varying potentials for 7.5 min. (**g**) Correlation of applied charge to cell fluorescence. Cells were mixed with the indicated concentrations of AS. A constant potential of + 0.7 V was applied for varying durations from 0 to 10 min (red), or the potential was varied from 0 to + 0.7 V and applied for 20 min (green). The linear regression line, calculated for all values whether varying time or varying potential, and R^2^ value are shown. (**d**), (**f**), and (**g**) show fluorescence after three hours of incubation normalized to OD_600_ and are reported as fold change relative to untreated cells. Schematics (**a**) and (**b**) were generated in Microsoft PowerPoint Version 2401. (**d**), (**e**), and (**f**) were generated in Matlab R2020a. (**c**) and (**g**) were generated in Microsoft Excel Version 2401.

A promising outlook in electrogenetic control is to turn gene expression “on” by simple application of an electronic cue^14^. To demonstrate, we added AS to reporter cells and subsequently applied an oxidizing potential. This differs from the oxidized AS induction described in the previous experiments, where a bolus addition of oxidized AS induced fluorescence. Instead, here we show direct electrogenetic control: the signal for inducing expression of the OxyRS-sfGFP reporter cells is the application of an oxidative potential to an electrode immersed in a growing cell culture, rather than bolus addition of an oxidized chemical inducer (Fig. 5a). The electrochemical half-cell setup was similar to the setup for oxidation of AS, but we used a 1:1 mix of reporter cell culture and AS diluted in PB to the desired concentration (Fig. 5b). When an oxidizing potential was applied to the mixture of cell culture and AS, an increase in fluorescence was observed. This was directly analogous to the results from bolus addition of oxidized AS to reporter cells (Fig. 5c vs Fig. 4b). Gene expression and fluorescence could be tuned by varying the concentration of AS added as well as the duration of the applied oxidative voltage (Fig. 5d). Higher AS concentrations and application of oxidizing potential for a longer duration increasingly inhibited cell growth (Fig. 5e), such that inducing reporter cells using this method requires optimizing AS concentration and applied charge to avoid altered cell growth. Varying the potential applied also provides significant power to modulate gene expression (Fig. 5f). Importantly, we found that the accumulated applied charge correlated linearly with GFP fluorescence in all cases, whether the parameter being varied was charge duration or potential (Fig. 5g). The correlation between charge and gene expression supports the argument that applied charge is the effective parameter controlling gene expression, such that normalizing gene expression to charge enables consistency for comparison across distinct experiments. That is, we show here that gene expression can be reliably tuned based on total accumulated charge, and either potential or charge duration can be adjusted for fine-tuned modulation of gene expression.

### An electronic switch: a duality in controlling gene expression

**Figure 6:**
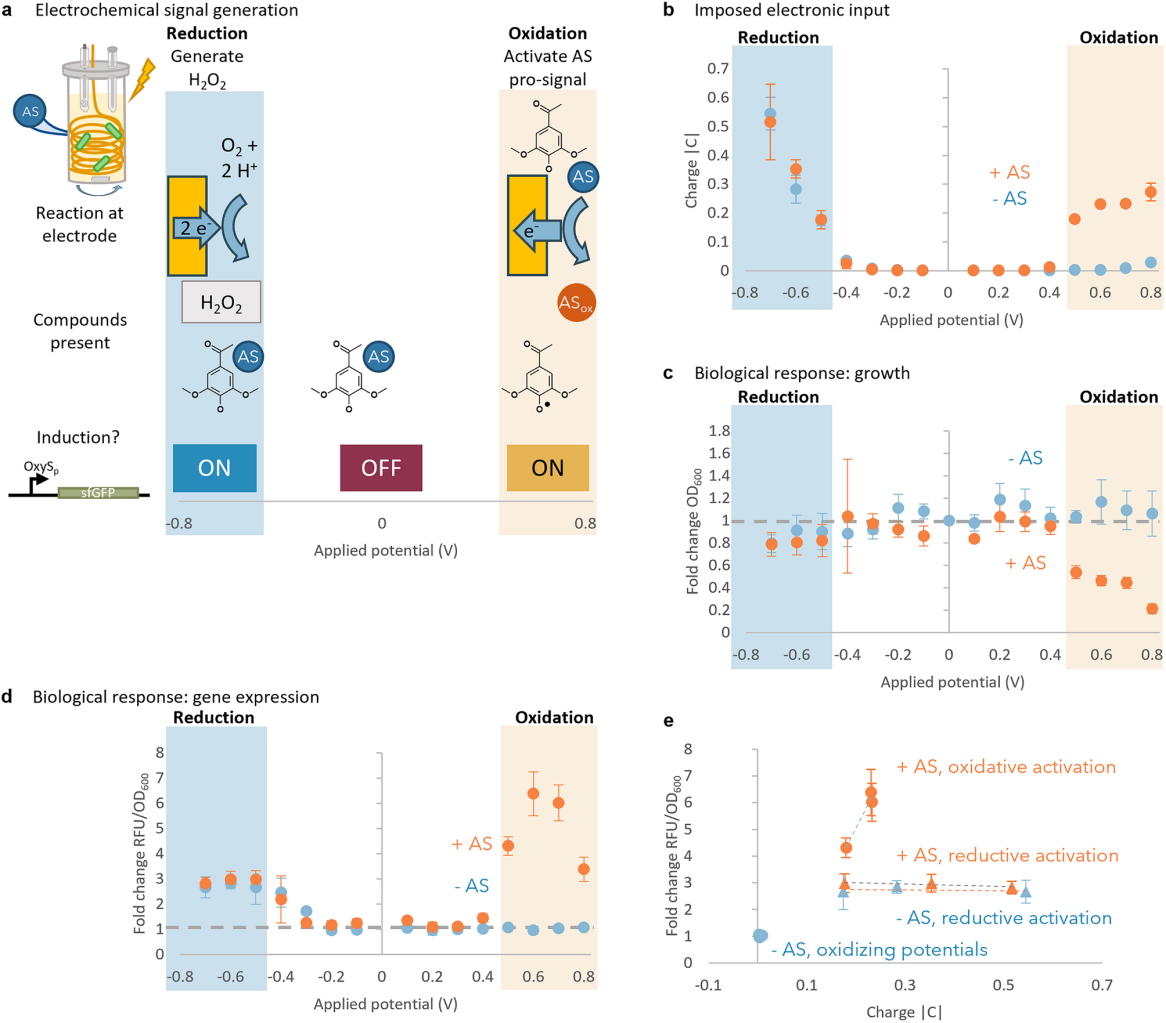
Biological responses (growth and gene expression) are elicited by electrochemical signals generated at distinct potential ranges. (**a**) Result of applying electronic signals to cells in the presence and absence of AS. Application of a reducing potential leads to H_2_O_2_ generation due to the oxidation–reduction reaction. Application of an oxidizing potential generates oxidized AS. Both H_2_O_2_ and oxidized AS induce gene expression via the OxyR regulon. (**b**) Cumulative applied charge, (**c**) OD_600_ after three hours of incubation normalized to untreated cells, and (**d**) fold fluorescence of POxyRS-sfGFP reporter cells after varying potentials are applied for 7.5 min in the absence or presence of 500 μM AS. Fluorescence is reported after three hours of incubation and is normalized to OD_600_, then the RFU/OD_600_ value is normalized to that of untreated cells. (**e**) Correlation of cumulative applied charge to gene expression for cells to which AS was added or omitted and reducing (− 0.5 to − 0.7) or oxidizing (+ 0.5 to + 0.7) potentials were applied. Linear regressions have a slope of 0.25 (− AS, reducing potentials), 0.81 (+ AS, oxidizing potentials), and 43.0 (+ AS, oxidizing potentials). Schematic (**a**) was generated using Microsoft PowerPoint Version 2401 and PubChem Sketcher V2.4. (**b**–**e**) were generated in Microsoft Excel Version 2401 and show the average of three biological replicates.

It is important to note that electrogenetic control of the *oxyS* promoter is also achieved using the 2-electron oxygen-reduction reaction (ORR) to convert dissolved oxygen into H_2_O_2_, thereby inducing OxyR-regulated gene expression by electronically generating one of its native signals^15,17,41^. The ORR is carried out at reducing potentials (− 0.5 V with a gold electrode ^18,41^) whereas oxidation of AS occurs at oxidizing potentials, and both H_2_O_2_ and oxidized AS are now shown to induce expression via OxyR (Fig. 6a). To verify induction of OxyRS-sfGFP reporters by electrode-generated H_2_O_2_ and to explore the extent to which one can use varied inputs, we applied potentials varying from + 0.8 to − 0.7 V to reporter cell cultures in the absence of any mediator. As expected, only application of the reducing potentials required for ORR (from − 0.5 to − 0.7 V^41^) accumulated charge (Fig. 6b), consistent with generation of H_2_O_2_ at the electrode^41^. Interestingly, when the same experiment was repeated in the presence of acetosyringone, charge accumulation was observed both at oxidizing potentials (+ 0.5 to + 0.8 V) and reducing potentials (− 0.5 to − 0.7 V, Fig. 6b). While most of the applied potentials resulted in comparable growth rates and final OD_600_ values, applying oxidizing potentials inhibited growth only in the presence of AS, consistent with earlier observations (Fig. 6c). Gene expression followed the same trends as the accumulated charge. Without AS present, only reducing potentials elicited fluorescence (“reductive activation”), whereas when AS was present, fluorescence was observed at both reducing and oxidizing potentials (“oxidative activation”) (Fig. 6d and Supplementary Fig. S3). Interestingly, induction by oxidation of AS led to higher fluorescence per unit charge (Fig. 6e). These results suggest that AS can facilitate gene expression at two distinct potential ranges: at oxidizing potentials, as oxidized AS induces the *oxyS* promoter; and at reducing potentials, as the presence of AS does not interfere with ORR-based H_2_O_2_ generation, allowing for induction of the *oxyS* promoter by H_2_O_2_.

### Dynamic electrogenetic programming

**Figure 7:**
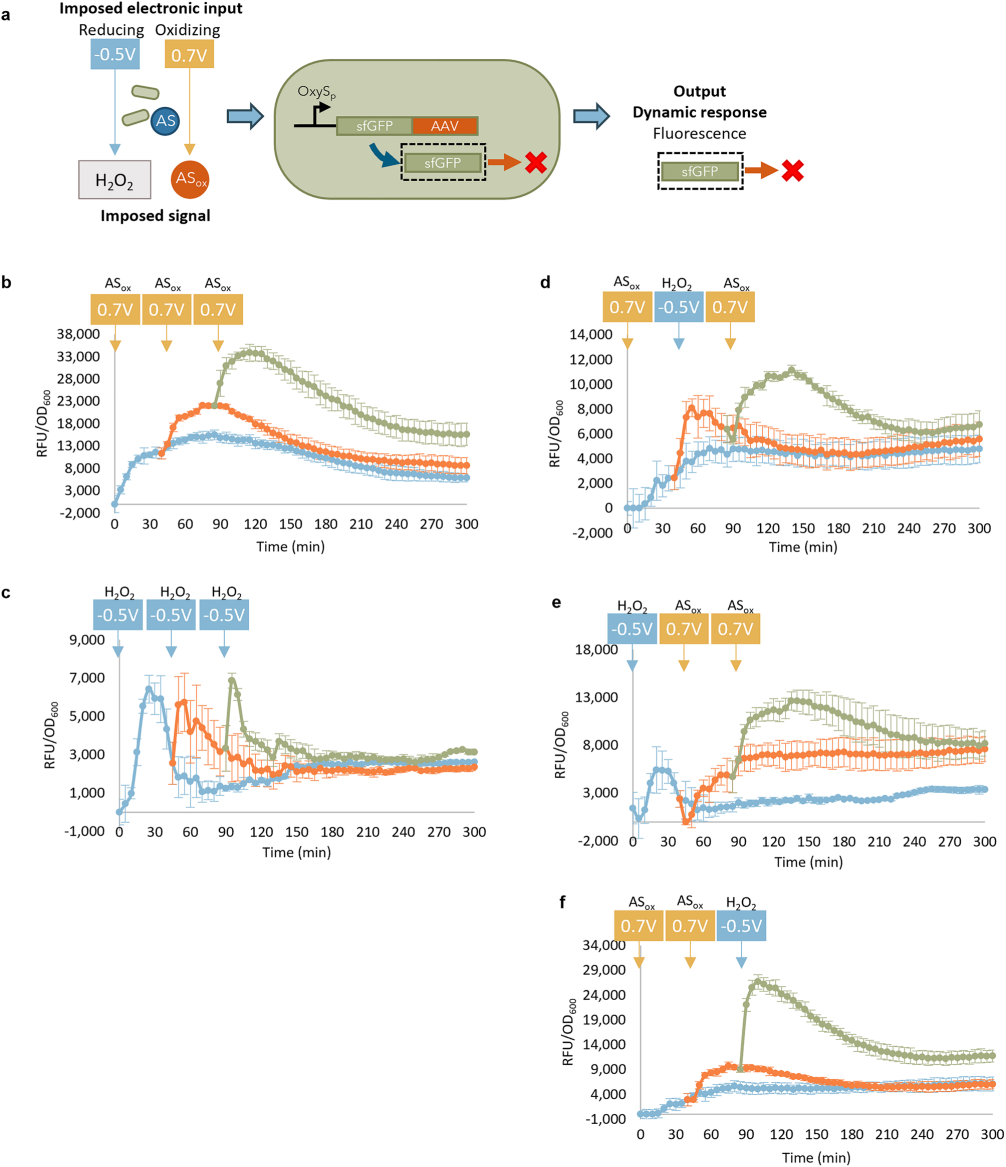
Electrochemically generated signals influence gene expression dynamics. (**a**) Scheme of electrochemical signals generated at reducing and oxidizing potentials in the presence of AS and reporter cells expressing degradation-tagged fluorophores. Fluorescence of POxyRS-sfGFP-AAV reporter cells, in the presence of 500 μM AS, after application of “pulses” of potential at the indicated voltage and time for 7.5 min. If the value of fluorescence difference between the sample and the untreated cell negative control was less than 5 RFU, it was denoted as 0 RFU. The applied potential “pulses” were (**b**) three oxidizing pulses, (**c**) three reducing pulses, (**d**) an oxidizing, reducing, then oxidizing pulse, (**e**) one reducing followed by two oxidizing pulses, or (**f**) two oxidizing pulses followed by a reducing pulse. Schematic (**a**) was generated using Microsoft PowerPoint Version 2401. (**b**–**f**) were generated in Microsoft Excel Version 2401.

To compare our acetosyringone-based electrogenetic approach with ORR-based H_2_O_2_ production, we mixed acetosyringone with OxyRS-sfGFP-AAV reporter cells and treated in varied electronic modalities by applying 5-min “pulses” of either oxidizing potential (+ 0.7 V) or reducing potential (− 0.5 V) and different combinations thereof (Fig. 7a). In Fig. 7b, we pulsed + 0.7 V in a repeated fashion for 5 min (− 160 ± 47 mC charge) every 45 min. It was readily evident that the added oxidizing potential repeatedly induced the cells, and interestingly, nearly equivalently at each step. The degradation-tagged sfGFP showed peaks in fluorescence followed by decreasing levels starting from 30 to 90 min after the pulse, back towards a still induced but lower level. With multiple applications of a reducing potential (− 0.5 V for 5 min, or + 191 ± 55 mC charge), we found sharp peaks followed by more rapid decay until all reached similar levels typically an hour after the peak in fluorescence (Fig. 7c). Subsequent tests with alternating oxidation and reduction pulses resulted in varied responses with different patterns, peaks, and decay times (Fig. 7d–f). Interestingly, in each case where + 0.7 V was applied, there was a relatively slower response (in both increase and decrease of signal), while in each case where − 0.5 V was applied, there was a faster, sharper increase in fluorescence (Fig. 7b–f). Also, we note that typically, the resultant steady state values were apparently additive, such that a culture with three pulses reached higher levels than those with two and these were seen higher than those with a single pulse, even without normalizing expression to OD_600_ (Supplementary Fig. S4). The dynamic trends resulting from the oxidation and reduction pulses were consistent across biological replicates (Supplementary Fig. S5). Hence, applying multiple pulses to the reporter cell culture over time yielded somewhat predictable and additive fluorescence outputs, demonstrating dynamic temporal control of gene expression based on the type (potential), duration, and timing of the electronic input.

## Discussion

Electronic control of gene expression expands our ability to program and manipulate cellular behavior. In this work, we demonstrate that redox electrogenetics can be facilitated by acetosyringone via transcriptional regulator OxyR and the *oxyS* promoter. OxyR has been described to be primarily activated by hydrogen peroxide and partially activated by certain S-nitrosothiols^33^ and decreases in the cellular thiol-disulfide ratio^47^. Oxidized acetosyringone, a phenolic plant-produced redox mediator, is reported here for the first time to be an inducer of OxyR. Importantly, this work introduces a new mechanism and electronic potential range for tunable electronic control of gene expression. We show that OxyR-mediated expression can be electronically induced both at reducing potentials (< − 0.5 V^17^) by electrode-generated H_2_O_2_, and at oxidative potentials (> 0.7 V) by oxidized acetosyringone (Fig. 6a). Induction of SoxR-mediated gene expression has previously been achieved at oxidative potentials only (> 0.3 V^14^), and the electrochemical configuration was based on two mediators—pyocyanin and ferricyanide/ferrocyanide—as well as the maintenance of an anaerobic environment. Acetosyringone-facilitated induction, on the other hand, provides a simple, tunable, and accessible electrogenetic scheme based on a single mediator that works in an aerobic environment, thus expanding the range over which electronic activation can be applied. In addition, because each of the described methods for electronic induction are accessed at distinct potential ranges (more negative than − 0.5 V, more positive than 0.3 V, more positive than 0.7 V), future work can multiplex electronic inputs by utilizing applied potential as a “knob” to selectively toggle expression of desired genes.

To our knowledge, this is the first demonstration that the redox signaling capabilities of acetosyringone can be harnessed to electronically actuate cellular function. Acetosyringone is produced by plants during wound stress and has been well studied for its signaling mechanism involving molecular recognition. Specifically, AS interacts with *vir* two-component receptor systems, inducing chemotaxis and virulence gene expression in *Agrobacterium* via the *vir* regulon^48^. AS can also act as a signaling molecule through another, distinct mechanism: it can interact through redox activity. The oxidized form of AS can be generated enzymatically^49^ or electrochemically^50^, and previous work suggested that oxidized AS could elicit stress responses in *Pseudomonas syringae*, upregulating expression of genes involved in metabolism, energy generation, and cell wall components and inducing a viable but not culturable (VBNC) state^51^. Our work builds upon such insights by demonstrating that oxidized AS, but not reduced AS, specifically interacts with the OxyR signal transduction pathway. In this capacity, AS serves as a “pro-signal”, wherein its redox-based messaging is conveyed when it is in the oxidized form. “Pro-signals” are consistent with the concept that redox signaling depends more on molecular activities than molecular structure^8^. While we showed that AS can act as a redox-state-dependent signal, we did not attempt to study the intracellular signal transduction mechanisms. As OxyR and its hydrogen peroxide-responsive homologs are widely conserved across many bacteria^32^, future applications of this work may involve sensing or actuation of other, rhizosphere-relevant, bacteria.

Interestingly, we observed a difference in the dynamics of the OxyR-regulated responses elicited by H_2_O_2_ and oxidized AS. Importantly, we show that a single promoter can be toggled using two distinct mechanisms simply by varying the type of potential applied to cells in the presence of AS. The imposed electronic input, or redox context, defines the duration and magnitude of the biological response, thus further demonstrating the importance of context for redox activity^52,53^. Both H_2_O_2_ and oxidized AS are signaling molecules present in the rhizosphere after plant infection. The difference in the dynamic responses to the two signaling molecules may have implications relative to the bacterial response to plant-produced oxidative stressors. We note further that physicochemical or spatial effects may arise from gradients projecting from the electrode to the solution^41,54^ (analogous to the plant and rhizosphere). Such gradients are a characteristic feature of redox signaling, and have been partially mitigated in electrogenetics by using gold-binding peptide surface display that serves to localize cells onto gold electrodes and the electrode-generated signal^30^ or by using an intermediate “transmitter” cell population that transduces a transient electrode-generated signal (H_2_O_2_) into a more sustained acyl homoserine lactone quorum sensing signal^41^.

We also found that by selectively tuning and applying oxidizing and reducing potentials in pulses, we could generate patterns of oscillating gene expression (Fig. 7). Oscillatory patterns are natively found in biology, including signaling molecules that are expressed in pulses^55^ and plant responses to multiple stresses^56^. As such, our electrochemical setup using acetosyringone could be used to recapitulate natural oscillatory activity. At the same time, biomanufacturing applications could involve synthetic strains to improve production of relevant molecules^57^, or activate a particular cellular response that is present for either a transient period or for a sustained duration depending on whether the inducer present is H_2_O_2_ or oxidized AS.

Understanding the signaling characteristics of oxidized acetosyringone provides new avenues for learning and building technologies for the soil rhizosphere as well as for engineered systems. Using the acetosyringone induction system, one could envision biosensors, cells and cell networks that collect new types of information about their local redox state wherein they are programmed to carry out designer functions (e.g., generate nutrients for root structures, degrade recalcitrant contaminants, or otherwise synthesize value-added products).

## Materials and methods

### Strains and plasmids

The strains, plasmids, and primers used in this work are listed in Table 1. Plasmid pOxyRS-sfGFP-AAV is derived from pOxyRS-sfGFP^38^ and incorporates a *ssrA* degradation tag encoding AANDENYLAAAV^45^ (“AAV”) at the 3ʹ end of sfGFP.

**Table 1:** Strains, plasmids, and primers used in this study.

	Description	Source
Strains		
* E. coli*		
NEB10-beta	*Δ(ara-leu) 7697 araD139 fhuA ΔlacX74 galK16 galE15 e14-ϕ80dlacZΔM15 recA1 relA1 endA1 nupG rpsL* (Str^R^) *rph spoT1 Δ(mrr-hsdRMS-mcrBC)*	New England Biolabs
W3110	K12 strain, wild type, *λ*-, F-, IN(*rrnD-rrnE*)1, *rph-1s*	Genetic Stock Center Yale University, New Haven, CT
ZK126	W3110 Δ*lacU169 tna-2*	^58^
SW102	ZK126 Δ*oxyRS*	^59^
Plasmids		
pOxyRS-sfGFP	pBR322, *oxyR* under constitutive *proD* promoter, sfGFP under *oxyS* promoter. Amp^R^	^38^
pOxyRS-sfGFP-AAV	pOxyRS-sfGFP derivative with *ssrA* degradation tag encoding AANDENYLAAAV (“AAV”) at 3ʹ end of sfGFP before stop codon. Amp^R^	This work

Primers	Sequence and purpose	Source
oxyR_F	gccagccgacgcttagc (for *oxyR* qPCR)	^60^
oxyR_R	aacatcacgcccagctcatc (for *oxyR* qPCR)	^60^
16S_rRNA_F	gttaatacctttgctcattga (for *E. coli* 16s rRNA qPCR)	^61^
16S_rRNA_R	accagggtatctaatcctgtt (for *E. coli* 16s rRNA qPCR)	^61^
katG_F	gccgatctacaacccgac (for *katG *qPCR)	This work
katG_R	gtagaagcagatgcccagg (for *katG *qPCR)	This work

### Cell culture

Plasmid pOxyRS-sfGFP was cloned into strain SW102 (ZK126 Δ*oxyRS*) and plasmid pOxyRS-sfgFP-AAV was cloned into NEB10-beta. All experiments involving these two plasmids were performed in the aforementioned strains, respectively. Cells were cultured at 37 °C with shaking at 250 rpm in an incubator or in a TECAN SPARK microplate reader. LB media was used for cloning and for growing 4–5 mL overnight cultures. For cell experiments, overnight cultures were diluted 100× into M9 media (1× M9 salts, 0.1 mM CaCl_2_, 2 mM MgSO_4_, 0.4% glucose, and 0.2% casamino acids) with the appropriate antibiotics and cultured until the OD_600_ reached ~ 0.4 before addition of inducer(s). Unless indicated, all other cell experiments used a 1:1 mixture of M9 media and 0.1 M phosphate buffer (PB, pH = 7.2) (with acetosyringone when indicated). For ampicillin-resistant plasmids (pOxyRS-sfGFP and pOxyRS-sfGFP-AAV), ampicillin was used at a concentration of 100 μg/mL. Microplate reader experiments were conducted using 96-well plates and 200 μL sample volume, and samples were measured in triplicate.

### Electrochemical setup

Electrochemical techniques were run using a CHI 6273 C electrochemical analyzer (CH Instruments). All electrochemical methods used a three-electrode setup in a 17 mm diameter glass vial with an Ag/AgCl reference electrode, platinum wire counter electrode, gold working electrode, and small stir bar. For cyclic voltammetry, a 2 mm gold standard working electrode (CH Instruments) was used, and potential was scanned from 0 to 0.7 V and back at a scan rate of 0.2 V/s with a 0.001 V sample interval and 1 × 10^–5^ A/V sensitivity. For routine oxidation of acetosyringone with a half-cell setup^41^, a 4.5 mL, 2 mM solution of AS (Sigma-Aldrich) in PB was prepared, and the electrochemical setup used a salt bridge as well as a 0.5 mm diameter gold wire (Sigma-Aldrich) approximately 75.4 cm long, coiled to fit in the vial. Using the amperometric i-t curve technique, a constant potential of 0.7 V was applied for 20 min with a 0.1 s sample interval and 1 × 10^–3^ A/V sensitivity. The potential and duration of applied charge were varied as described in the experimental section. For oxidation of cell culture, the same setup was used, but with 2.4 mL of a 1:1 mixture of cell culture (OD_600_ of 0.4) and either PB only or AS and PB. AS concentration, potential, and duration of applied charge were varied as described in the experimental section. For application of “pulses” of potential, a 2.4 mL of a 1:1 mixture of cell culture (OD_600_ of 0.4) and PB with AS (final AS concentration of 500 μM) was prepared and the indicated potential was applied for 7.5 min. 50 μL samples were extricated in triplicate, diluted to 200 μL with equal volumes of M9 media and PB, and placed in a 96-well plate for incubation and measurement in the plate reader. Simultaneously, the remaining cell solution was incubated in a shaking incubator at the same temperature and shaking speed. After 45 min of incubation, the cell solution was replenished with 150 μL of a mixture of M9, PB, and AS to the same concentration as the initial setup. The same steps were repeated for a second and third “pulse” of potential.

### Spectrophotometric and fluorometric readings

Absorbance and fluorescence were measured using a TECAN Spark microplate reader. Absorbance spectra of AS were measured with a clear 96-well plate. For cell experiments, 200 μL samples were loaded in triplicate in a black 96-well plate with a clear bottom. The plate was placed in a humidity cassette in the microplate reader, set at 37 °C with shaking, and OD_600_ and sfGFP fluorescence (485 nm excitation and 520 nm emission) were continually measured. Fluorescence (relative fluorescence units, or RFU) and absorbance units were normalized by subtracting measurements of the PB/M9 blank sample. Fluorescence was further normalized by dividing by OD_600_ (RFU/OD_600_); when indicated, RFU/OD_600_ was normalized to the RFU/OD_600_ of untreated cell samples (Fold change relative to untreated). Background fluorescence from oxidized AS at the concentrations utilized was negligible (Supplementary Fig. S6), so fluorescence was normalized by subtraction of the PB/M9 blank sample rather than subtraction of oxidized AS samples.

### qPCR assay

6 mL of NEB10β cells were grown to an OD_600_ of 0.4 and induced with PB (negative control), 100 μM H_2_O_2_, or 0.75 mM of oxidized AS. After 30 min incubation, 1 mL of cells was pelleted in triplicate for biological replicates. RNA was isolated with the TRIzol^®^ Max™ Bacterial RNA Isolation Kit (ThermoFisher) according to the manufacturer’s protocol and treated with DNAse I (New England Biolabs). Quantitative real time PCR was run on Applied Biosystems QuantStudio 7 Flex (ThermoFisher) using ~ 200 ng RNA, 1.5 μM of the qPCR primers listed in Table 1, and the Power SYBR^®^ Green PCR RT-PCR mix following the manufacturer’s protocol (ThermoFisher). Gene expression fold change averages and standard deviations were calculated using ΔΔCT relative to *E. coli* 16s rRNA as an internal control and compared to the negative control of untreated cells.

### CFU count assay

250 μL of NEB10β cells grown to an OD_600_ of 0.4 were transferred to microcentrifuge tubes and treated with PB (negative control), AS, or oxidized AS. Tubes were incubated for 1 h at 37 °C, then four 10× serial dilutions were prepared in PB. 5 μL of each dilution was pipetted onto an LB agar plate in triplicate and incubated overnight. Individual colonies were counted at the highest dilution and colony forming units (CFU) were determined based on the dilution factor.

### Supplementary Information

Supplementary Information.

## Data Availability

The datasets generated during and/or analyzed during the current study are available from the corresponding author on reasonable request.

## References

[1] Parlak O, Turner AP (2016). Switchable bioelectronics. Biosens. Bioelectron..

[2] Mansouri M, Fussenegger M (2022). Electrogenetics: Bridging synthetic biology and electronics to remotely control the behavior of mammalian designer cells. Curr. Opin. Chem. Biol..

[3] Akyildiz IF, Pierobon M, Balasubramaniam S, Koucheryavy Y (2015). The internet of bio-nano things. IEEE Commun. Mag..

[4] Atkinson JT, Chavez MS, Niman CM, El-Naggar MY (2023). Living electronics: A catalogue of engineered living electronic components. Microb. Biotechnol..

[5] Tseng C-P, Silberg JJ, Bennett GN, Verduzco R (2020). 100th anniversary of macromolecular science viewpoint: Soft materials for microbial bioelectronics. ACS Macro Lett..

[6] Chin JW (2006). Modular approaches to expanding the functions of living matter. Nat. Chem. Biol..

[7] Weber W (2007). A synthetic time-delay circuit in mammalian cells and mice. Proc. Natl. Acad. Sci..

[8] Jones DP, Sies H (2015). The redox code. Antioxid. Redox Signal..

[9] Lee I-G, Lee B-J (2021). How bacterial redox sensors transmit redox signals via structural changes. Antioxidants.

[10] Kim E (2019). Redox is a global biodevice information processing modality. Proc. IEEE.

[11] Demple B (1996). Redox signaling and gene control in the *Escherichia coli* soxRS oxidative stress regulon—a review. Gene.

[12] Storz G, Tartaglia LA, Ames BN (1990). The oxyR regulon. Antonie Van Leeuwenhoek.

[13] Huang J, Xue S, Buchmann P, Teixeira AP, Fussenegger M (2023). An electrogenetic interface to program mammalian gene expression by direct current.. Nat. Metab..

[14] Tschirhart T (2017). Electronic control of gene expression and cell behaviour in *Escherichia coli* through redox signalling. Nat. Commun..

[15] Bhokisham N (2020). A redox-based electrogenetic CRISPR system to connect with and control biological information networks. Nat. Commun..

[16] Melchionna M, Fornasiero P, Prato M (2019). The rise of hydrogen peroxide as the main product by metal-free catalysis in oxygen reductions. Adv. Mater..

[17] Stephens K, Zakaria FR, VanArsdale E, Payne GF, Bentley WE (2021). Electronic signals are electrogenetically relayed to control cell growth and co-culture composition. Metab. Eng. Commun..

[18] VanArsdale E (2022). Electrogenetic signaling and information propagation for controlling microbial consortia via programmed lysis.. Biotechnol. Bioeng..

[19] VanArsdale E (2020). A coculture based tyrosine-tyrosinase electrochemical gene circuit for connecting cellular communication with electronic networks. ACS Synth. Biol..

[20] Million M, Raoult D (2018). Linking gut redox to human microbiome. Hum. Microbiome J..

[21] Fuller AW (2017). Redox-mediated quorum sensing in plants. PLoS ONE.

[22] Schieber M, Chandel NS (2014). ROS function in redox signaling and oxidative stress. Curr. Biol..

[23] Reczek CR, Chandel NS (2015). ROS-dependent signal transduction. Curr. Opin. Cell Biol..

[24] Ushio-Fukai M (2009). Compartmentalization of redox signaling through NADPH oxidase–derived ROS. Antioxid. Redox Signal..

[25] Biswas S, Chida AS, Rahman I (2006). Redox modifications of protein–thiols: Emerging roles in cell signaling. Biochem. Pharmacol..

[26] Panieri E, Santoro MM (2015). ROS signaling and redox biology in endothelial cells. Cell. Mol. Life Sci..

[27] Paulsen CE, Carroll KS (2013). Cysteine-mediated redox signaling: Chemistry, biology, and tools for discovery. Chem. Rev..

[28] Kim E (2017). Redox probing for chemical information of oxidative stress. Anal. Chem..

[29] Motabar D, Li J, Payne GF, Bentley WE (2021). Mediated electrochemistry for redox-based biological targeting: Entangling sensing and actuation for maximizing information transfer. Curr. Opin. Biotechnol..

[30] Terrell JL (2021). Bioelectronic control of a microbial community using surface-assembled electrogenetic cells to route signals. Nat. Nanotechnol..

[31] Pomposiello PJ, Demple B (2001). Redox-operated genetic switches: The SoxR and OxyR transcription factors. Trends Biotechnol..

[32] Chiang SM, Schellhorn HE (2012). Regulators of oxidative stress response genes in *Escherichia coli* and their functional conservation in bacteria. Arch. Biochem. Biophys..

[33] Zheng M, Åslund F, Storz G (1998). Activation of the OxyR transcription factor by reversible disulfide bond formation. Science.

[34] Blount JW, Masoud S, Sumner LW, Huhman D, Dixon RA (2002). Over-expression of cinnamate 4-hydroxylase leads to increased accumulation of acetosyringone in elicited tobacco cell-suspension cultures. Planta.

[35] Li J (2020). Mediated electrochemistry to mimic biology's oxidative assembly of functional matrices. Adv. Funct. Mater..

[36] Bhattacharya A, Sood P, Citovsky V (2010). The roles of plant phenolics in defence and communication during Agrobacterium and Rhizobium infection. Mol. Plant Pathol..

[37] Baker CJ (2014). Characterization of apoplast phenolics: In vitro oxidation of acetosyringone results in a rapid and prolonged increase in the redox potential. Physiol. Mol. Plant Pathol..

[38] Li J (2021). Interactive materials for bidirectional redox-based communication. Adv. Mater..

[39] Engström P, Zambryski P, Van Montagu M, Stachel S (1987). Characterization of *Agrobacterium tumefaciens* virulence proteins induced by the plant factor acetosyringone. J. Mol. Biol..

[40] Lohrke S, Nechaev S, Yang H, Severinov K, Jin S (1999). Transcriptional activation of *Agrobacterium tumefaciens* virulence gene promoters in *Escherichia coli* requires the *A. tumefaciens* RpoA gene, encoding the alpha subunit of RNA polymerase. J. Bacteriol..

[41] VanArsdale E (2022). Electrogenetic signal transmission and propagation in coculture to guide production of a small molecule, tyrosine. ACS Synth. Biol..

[42] Pardo I, Chanagá X, Vicente AI, Alcalde M, Camarero S (2013). New colorimetric screening assays for the directed evolution of fungal laccases to improve the conversion of plant biomass. BMC Biotechnol..

[43] Gordonov T (2014). Electronic modulation of biochemical signal generation. Nat. Nanotechnol..

[44] Szatmári Á (2021). A pattern-triggered immunity-related phenolic, acetosyringone, boosts rapid inhibition of a diverse set of plant pathogenic bacteria. BMC Plant Biol..

[45] Andersen JB (1998). New unstable variants of green fluorescent protein for studies of transient gene expression in bacteria. Appl. Environ. Microbiol..

[46] Virgile C (2018). Engineering bacterial motility towards hydrogen-peroxide. PLoS ONE.

[47] Åslund F, Zheng M, Beckwith J, Storz G (1999). Regulation of the OxyR transcription factor by hydrogen peroxide and the cellular thiol—disulfide status. Proc. Natl. Acad. Sci..

[48] Lai EM (2006). Proteomic analysis of *Agrobacterium tumefaciens* response to the vir gene inducer acetosyringone. Proteomics.

[49] Camarero S, Ibarra D, Martínez MJ, Martínez AT (2005). Lignin-derived compounds as efficient laccase mediators for decolorization of different types of recalcitrant dyes. Appl. Environ. Microbiol..

[50] Díaz-González M, Vidal T, Tzanov T (2011). Phenolic compounds as enhancers in enzymatic and electrochemical oxidation of veratryl alcohol and lignins. Appl. Microbiol. Biotechnol..

[51] Postnikova OA, Shao J, Mock NM, Baker CJ, Nemchinov LG (2015). Gene expression profiling in viable but nonculturable (VBNC) cells of *Pseudomonas syringae* pv. syringae. Front. Microbiol..

[52] Chun K (2021). Parsed synthesis of pyocyanin via co-culture enables context-dependent intercellular redox communication. Microb. Cell Fact..

[53] Kim E (2014). Context-dependent redox properties of natural phenolic materials. Biomacromolecules.

[54] Chun K, VanArsdale E, May E, Payne GF, Bentley W (2024). Assessing electrogenetic activation via a network model of biological signal propagation. Front. Syst. Biol..

[55] Purvis JE, Lahav G (2013). Encoding and decoding cellular information through signaling dynamics. Cell.

[56] Husson O (2021). Soil and plant health in relation to dynamic sustainment of Eh and pH homeostasis: A review. Plant Soil.

[57] Benzinger D, Ovinnikov S, Khammash M (2022). Synthetic gene networks recapitulate dynamic signal decoding and differential gene expression. Cell Syst..

[58] Connell N, Han Z, Moreno F, Kolter R (1987). An *E. coli* promoter induced by the cessation of growth. Mol. Microbiol..

[59] Wang S (2021). A redox-based autoinduction strategy to facilitate expression of 5xCys-tagged proteins for electrobiofabrication. Front. Microbiol..

[60] Rodríguez-Rojas A (2020). Non-lethal exposure to H_2_O_2_ boosts bacterial survival and evolvability against oxidative stress. PLoS Genet..

[61] Malinen E, Kassinen A, Rinttila T, Palva A (2003). Comparison of real-time PCR with SYBR Green I or 5′-nuclease assaysand dot-blot hybridization with rDNA-targeted oligonucleotide probes in quantificationof selected faecal bacteria. Microbiology.

