# Supplementary material for: Redox active plant phenolic, acetosyringone, for electrogenetic signaling
Source: Sci Rep. 2024 Apr 26;14:9666. doi: 10.1038/s41598-024-60191-7 (PMC11053109; doi:10.1038/s41598-024-60191-7)
Supplement: Supplementary file 1 — Supplementary Information. [file 41598_2024_60191_MOESM1_ESM.pdf]

Redox Active Plant Phenolic, Acetosyringone, for Electrogenetic Signaling

Fauziah Rahma Zakaria[1,2,3], Chen-Yu Chen[1,2,3], Jinyang Li[1,2,3,4], Sally Wang[1,2,3], Gregory F. Payne[2,3*], William E. Bentley[1,2,3*]

Supplementary Information

[1]Fischell Department of Bioengineering, University of Maryland, College Park, Maryland, United States

[2]Institute for Bioscience and Biotechnology Research, Rockville, Maryland, United States

[3]Robert E. Fischell Institute for Biomedical Devices, University of Maryland, College Park, Maryland, United States

[4]Division of Biology and Biological Engineering, California Institute of Technology, Pasadena, California, United States

a

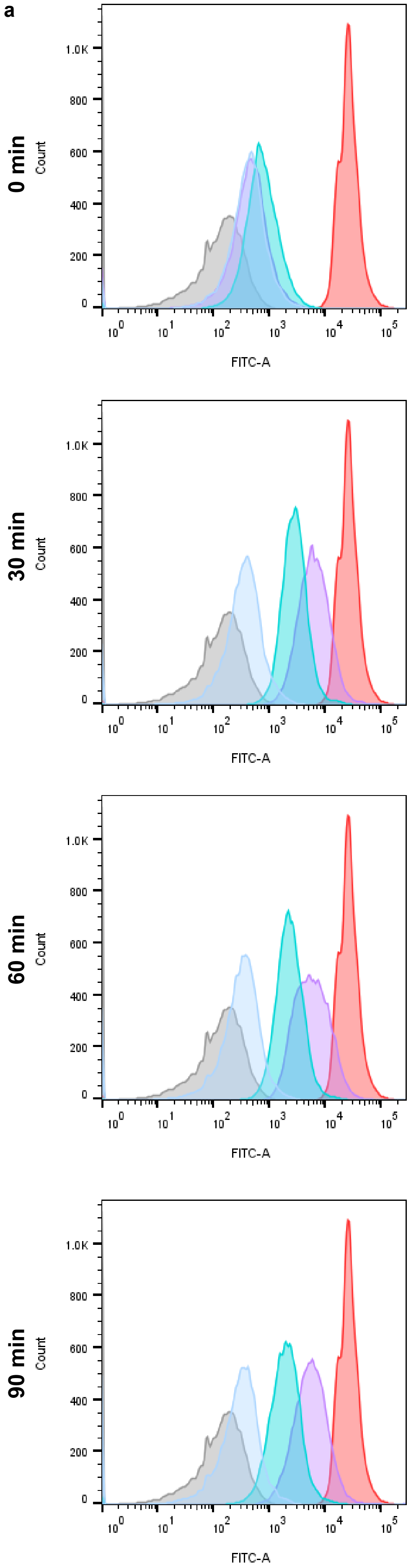

b

**Sample**		Cells | Frequency of Parent	Cells | Count	Cells | **Mean** (FITC-A)	Cells | **Median** (FITC-A)
Negative: DH5α (OD_{600} ~ 0.5)		99.4 %	19,887	143	103
Positive: DH5α-sfGFP (OD_{600} ~ 0.5)		97.9 %	19,588	27,270	24,435
0 min	OxyRS-sfGFP	99.5 %	19,906	559	411
	OxyRS-sfGFP + H_2O_2	98.3 %	19,656	946	699
	OxyRS-sfGFP + oxidized AS	99.6 %	19,927	598	438
30 min	OxyRS-sfGFP	99.3 %	19,857	452	342
	OxyRS-sfGFP + H_2O_2	98.8 %	19,767	3,205	2,677
	OxyRS-sfGFP + oxidized AS	99.5 %	19,906	7,802	5,891
60 min	OxyRS-sfGFP	99.6 %	19,910	409	313
	OxyRS-sfGFP + H_2O_2	99.5 %	19,896	2,681	2,176
	OxyRS-sfGFP + oxidized AS	99.8 %	19,963	7,715	5,377
90 min	OxyRS-sfGFP	99.6 %	19,910	388	292
	OxyRS-sfGFP + H_2O_2	99.7 %	19,935	2,298	1,857
	OxyRS-sfGFP + oxidized AS	99.6 %	19,929	7,309	5,248

Supplementary Figure S1 Fluorescence-Activated Cell Sorting (FACS) data to demonstrate induction by oxidized AS. OxyRS-sfGFP reporter cells were grown to mid-log and treated with PB (untreated control), 100 μM H_2O_2, or 750 μM oxidized AS. 0, 30, 60, and 90 minutes after treatment, cells were pelleted and fixed with 4% paraformaldehyde. For negative and positive controls, DH5α and DH5α-sfGFP (constitutive sfGFP expression), respectively, were resuspended from overnight cultures to an OD_{600} of around 0.5 and fixed. **a** Histograms and **b** statistics from FACS analysis (generated using FlowJo) are shown for the reporter cells at each time point, alongside the positive and negative DH5α controls.

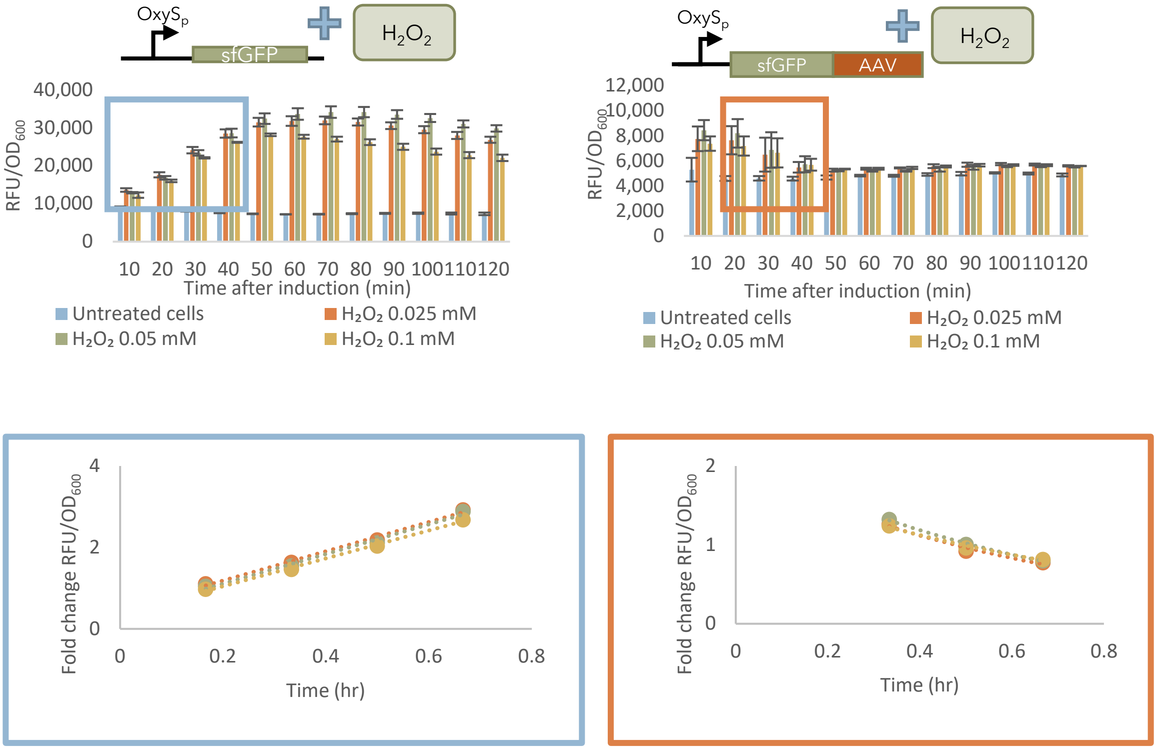

<u>Supplementary Figure S2</u> Figures **4a** and **4c** (top) are reproduced to highlight the regions used to calculate fluorophore expression and degradation rates (bottom).

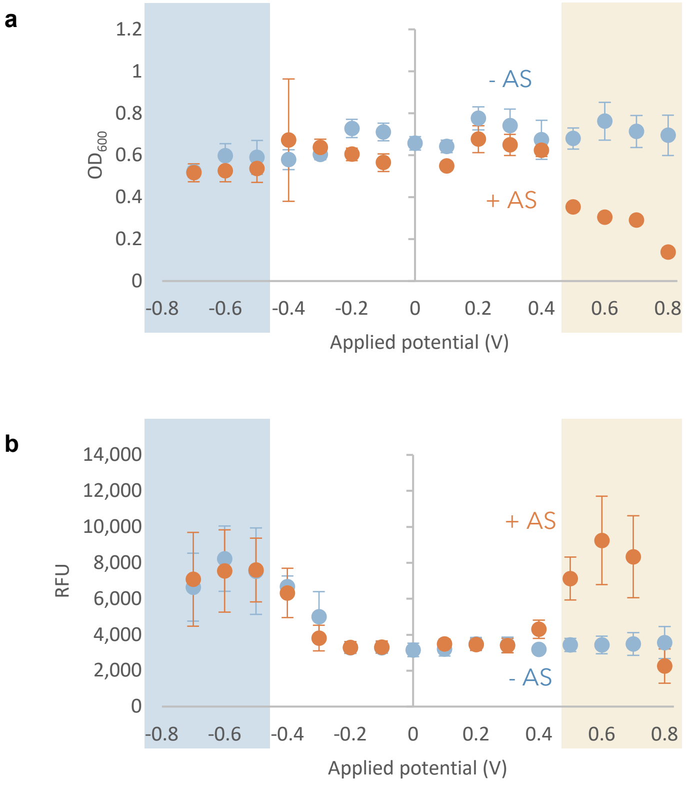

<u>Supplementary Figure S3</u> (**a**) OD_{600} and (**b**) fold fluorescence of POxyRS-sfGFP reporter cells after varying potentials are applied for 7.5 minutes in the absence or presence of 500 μM AS. Values are taken after three hours of incubation.

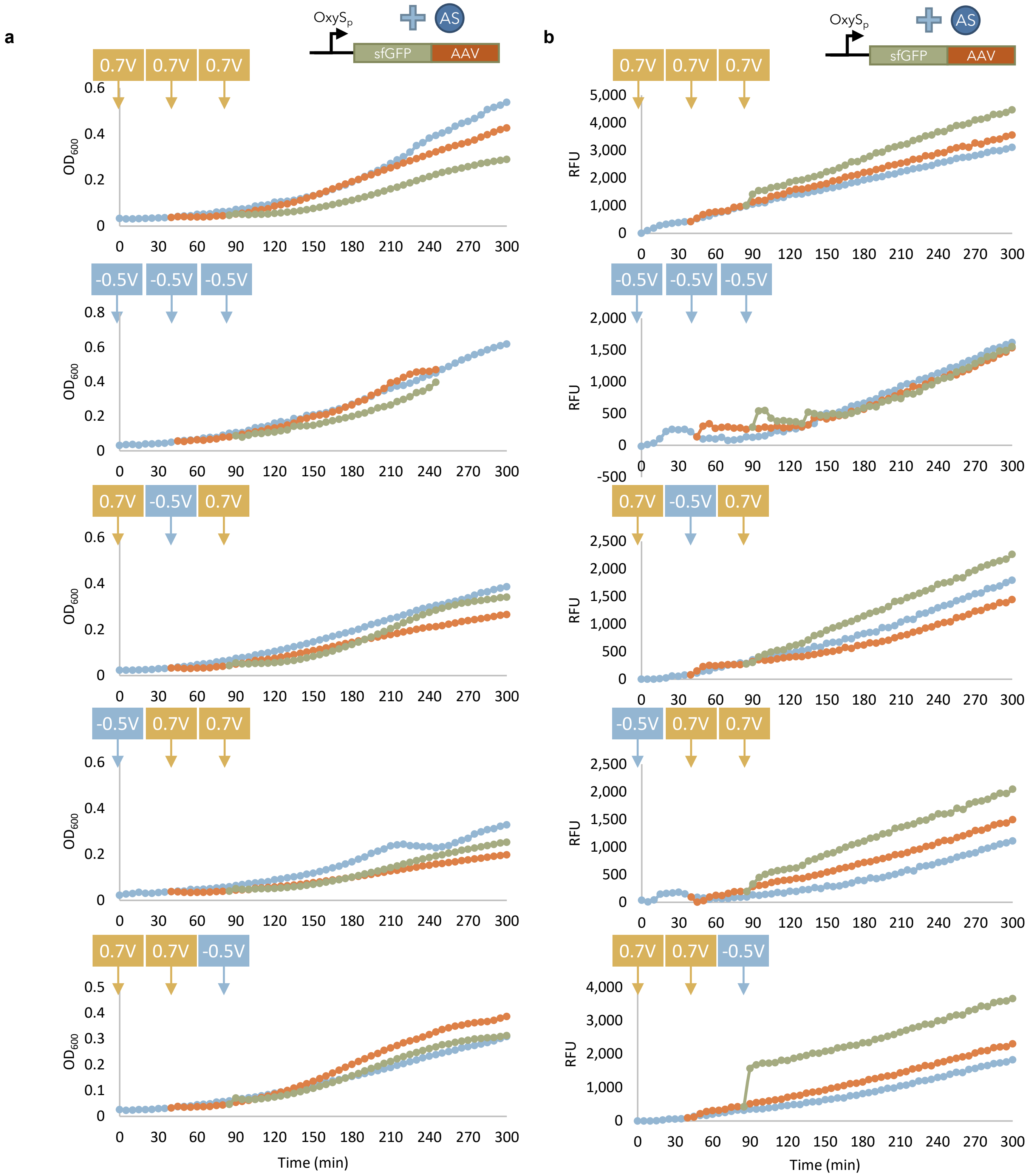

Supplementary Figure S4 (**a**) OD_{600} and (**b**) fluorescence of POxyRS-sfGFP-AAV reporter cells, in the presence of 500 μM AS, after application of "pulses" of potential at the indicated voltage and time for 7.5 minutes. If the value of fluorescence difference between the sample and the untreated cell negative control was less than 5 RFU, it was denoted as 0 RFU. Plots from top to bottom correspond to **Figures 7b-7f**.

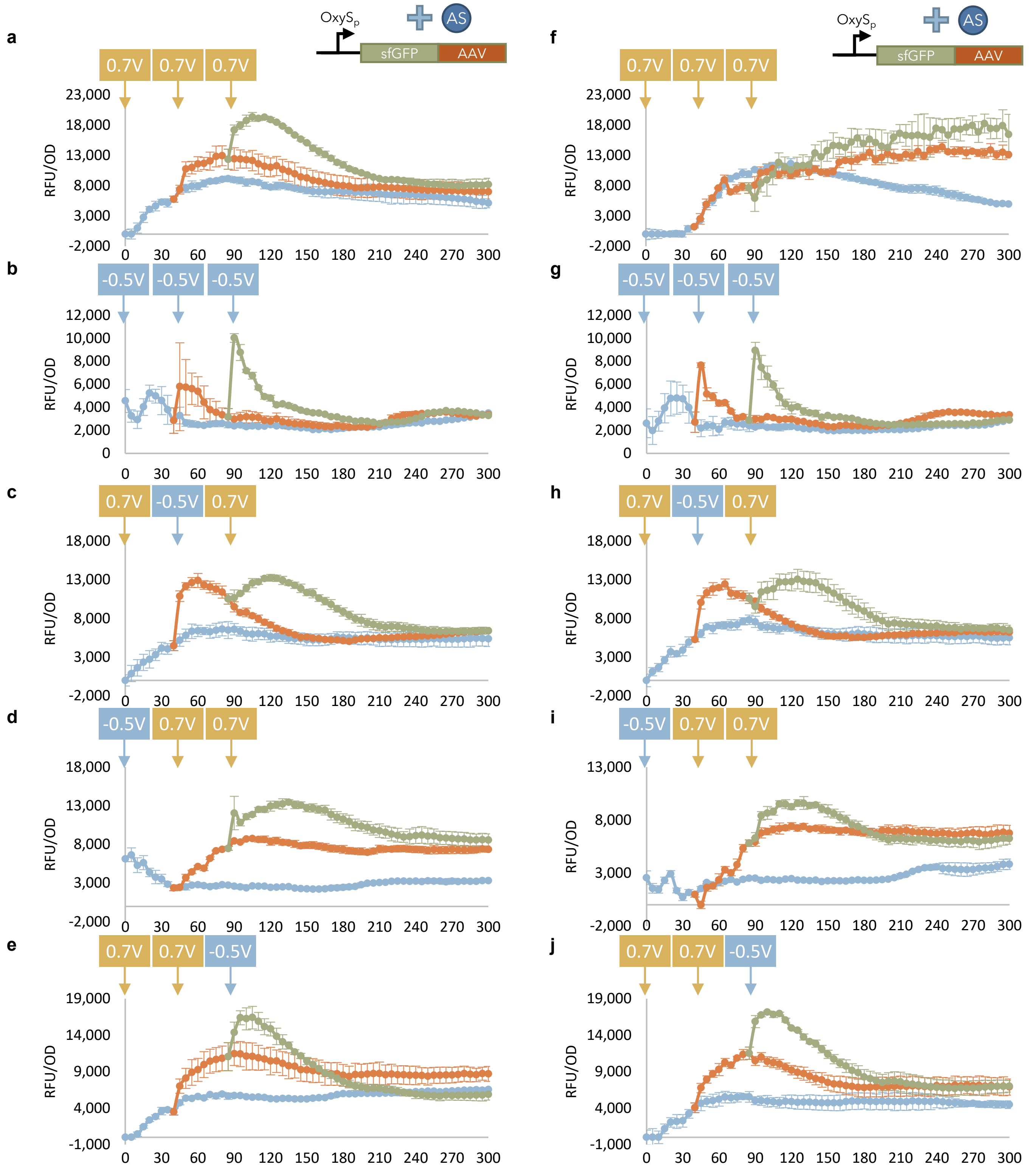

Supplementary Figure S5 Biological replicates of the data from **Fig. 7b-7f**. Plots from top to bottom correspond to **Figures 7b-7f** and show the fluorescence of POxyRS-sfGFP-AAV reporter cells, in the presence of 500 μM AS, after application of "pulses" of potential at the indicated voltage and time for 7.5 minutes: (**a**) & (**f**) three oxidizing pulses, (**c**) & (**g**) three reducing pulses, (**d**) & (**h**) an oxidizing, reducing, then oxidizing pulse, (**e**) & (**i**) one reducing followed by two oxidizing pulses, or (**f**) & (**j**) two oxidizing pulses followed by a reducing pulse. Data from (**a**)-(**e**), (**f**)-(**j**), and **Fig. 7b-7f** were each collected on separate days.

a

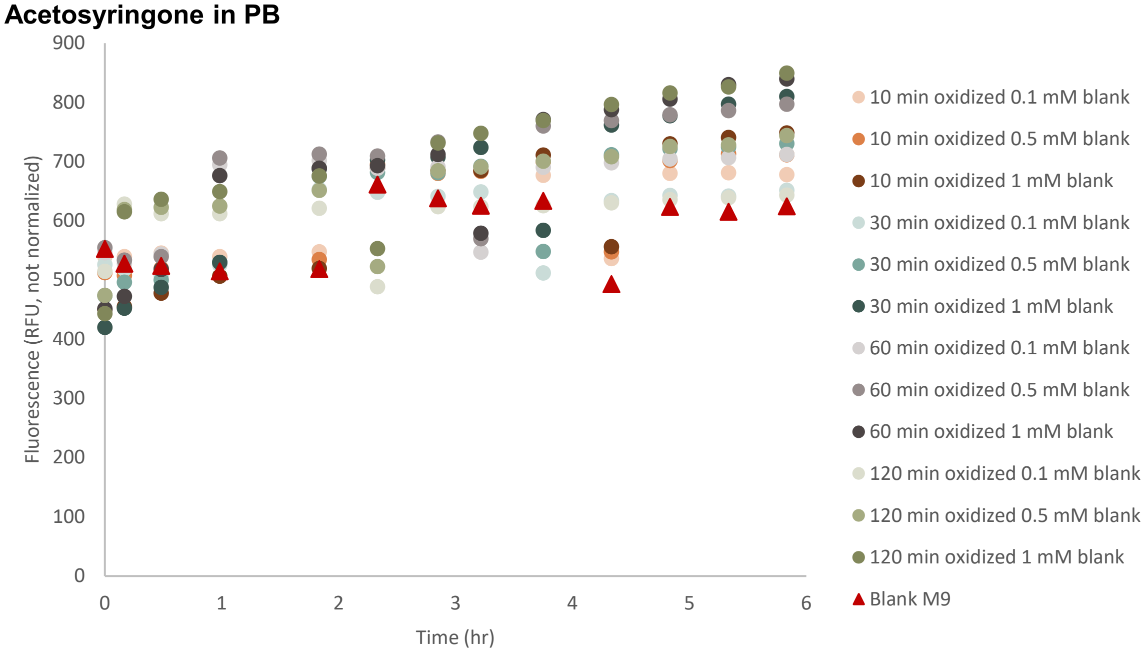

b

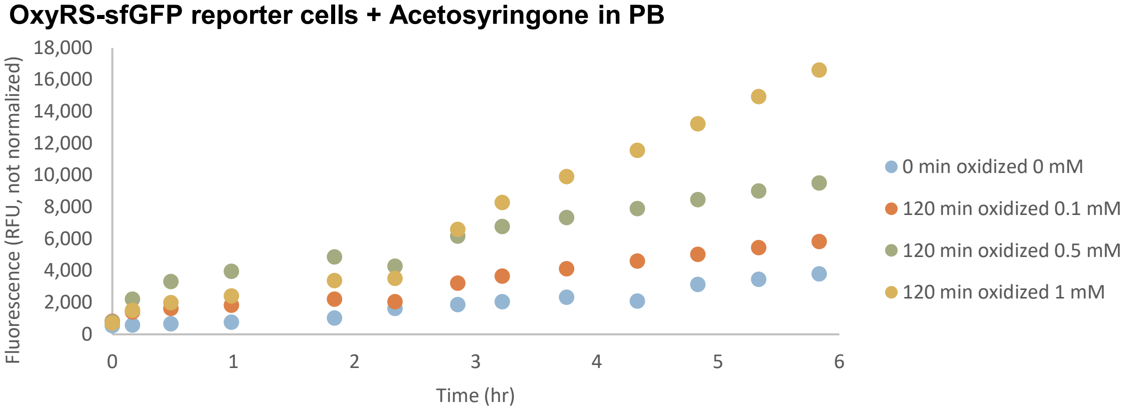

c

Length of time oxidized	Oxidized AS concentration							
	0 (M9 + PB Blank)		0.1 mM		0.5 mM		1 mM	
	RFU (avg)	St. dev	RFU (avg)	St. dev	RFU (avg)	St. dev	RFU (avg)	St. dev
	625.5	3.5						
10 min			683.0	14.1	683.5	0.7	685.0	18.4
30 min			648.5	6.4	692.0	8.5	724.0	2.8
60 min			547.0	5.7	569.5	4.9	578.5	36.1
120 min			623.5	0.7	690.5	16.3	747.5	4.9

Supplementary Figure S6 Acetosyringone was oxidized for different durations of time. This figure uses data from the experiment performed in Figure 3e, and values from triplicate wells were averaged. (**a**) Fluorescence of AS, oxidized for different durations of time, and diluted to different concentrations in M9. Raw fluorescence values are shown. (**b**) Fluorescence measured after AS, oxidized for different durations of time, was mixed with OxyRS-sfGFP reporter cells. Raw fluorescence values are shown. (**c**) Fluorescence values (average and standard deviation) at the 3.2-hour time point from the data in Supplementary Figure S5a.

Supplementary Tables

Inducer		sfGFP expression rate	sfGFP-AAV degradation rate
Inducer	Concentration (mM)		
		Zeroth-order	First-order
		Units: (RFU/OD_{600} fold change * hr^{-1}) ± standard error	Units: (RFU/OD_{600} fold change * hr^{-1}) ± standard error
H_2O_2	0.025	3.598 ± 0.215	-1.479 ± 0.254
	0.05	3.635 ± 0.261	-1.515 ± 0.076
	0.1	3.415 ± 0.151	-1.264 ± 0.152
Oxidized AS	0.25	1.007 ± 0.133	0.132 ± 0.059
	0.5	2.366 ± 0.407	-0.021 ± 0.061
	0.75	3.864 ± 0.789	-0.480 ± 0.048

Supplementary Table S1 Expression (synthesis) and degradation rates of sfGFP expressed by OxyRS-sfGFP or OxyRS-sfGFP-AAV reporter cells treated with each inducer. Expression rates were calculated as a zeroth-order maximum rate from linear regression of sfGFP fluorescence (normalized to untreated as fold change RFU/OD_{600}). Based on the region of increasing fluorescence, sfGFP expression rates for H_2O_2-treated cells were calculated from 10 to 40 minutes after induction, while rates for oxidized AS-treated cells were calculated from 20 to 40 minutes after induction.. Degradation rates were calculated as a first-order decay rate from exponential regression (b in $y = a * e^{bx}$) of sfGFP-AAV fluorescence (normalized to untreated as fold change RFU/OD_{600}). Based on the region of declining fluorescence, sfGFP-AAV degradation rates for H_2O_2-treated cells were calculated from 20 to 40 minutes after induction, while degradation rates for oxidized AS-treated cells were calculated from 70 to 100 minutes after induction. Standard errors of regression are reported.
